# Colored Polymeric Nanofiber Loaded with Minoxidil Sulphate as Beauty Coverage and Restoring Hair Loss

**DOI:** 10.1038/s41598-020-60863-0

**Published:** 2020-03-05

**Authors:** Fadilah Sfouq Aleanizy, Fulwah Yahya Alqahtani, Hamad M. Alkahtani, Bushra Alquadeib, Esraa K. Eltayeb, Amal Aldarwesh, Hosam G. Abdelhady, Ibrahim A. Alsarra

**Affiliations:** 1 Department of Pharmaceutics, College of Pharmacy, King Saud University, P.O. Box 22452, Riyadh, 11495 Saudi Arabia; 2 Department of Pharmaceutical Chemistry, College of Pharmacy, King Saud University, P.O. Box 2457, Riyadh, 11451 Saudi Arabia; 3 Eugene Applebaum College of Pharmacy and Health Sciences, Wayne State University, 42 W. Warren Ave., Detroit, MI USA; 4 Department of Optometry, College of Applied Medical Sciences, King Saud University, P.O. Box 22452, Riyadh, 11495 Saudi Arabia

**Keywords:** Nanobiotechnology, Health care

## Abstract

Polymeric nanofibers fabricated by electrospinning either blank (PVA) or loaded with minoxidil sulphate have yielded optimum fibers with an average diameter 273 nm, and 511 nm, respectively. Thermal analysis of nanofibers indicated no chemical interaction. The NMR spectrum confirmed stability of nanofiber as there were no interactions between functional groups. Prepared nanofibers showed a 47.4% encapsulation efficiency and 73% yield. *In vitro* drug release of minoxidil sulphate from nanofiber exhibited an initial burst release followed by a slower release pattern. Stability studies revealed that minoxidil nanofiber was stable if stored at room temperature and protected from light with only loss of 9.6% of its nominal concentration within 6 months. As a result, the prepared solid/colored formula serves as an ideal formulation for such instable drug in liquid formula taking the advantage of the attractiveness of beauty colored coverage, and the simple, and non-tousled application.

## Introduction

Electrospund nanofibers is widely used in a number of applications owing to their distinctive properties, such as high surface to volume ratio, high absorbency, tunable physicomechanical properties, easy adjustment of polymeric solutions and process parameters to produce anticipated fiber morphology and mechanical properties, and broad variety of naturally occurring and synthetic polymers that can be electrospun into nanofibers^1–3^. Regarding biological perspective, the human tissues and organs consists of native extracellular matrix (ECM) network of micro/nano-scaled fibers containing protein and glycosaminoglycan that offer support to resident cells and control cellular activities^1^. Owing to the resemblance between the fibrous architecture of native ECM and electrospun nanofibers, nanofibers were exploited in a number of biomedical applications; namely tissue-engineering, drug delivery, cosmetics, as well as biosensors^1,2,4^.

Minoxidil, applied topically, is widely used for the treatment of androgenic alopecia^5^, a hair loss in men and women^6,7^. It was reported that about 40% of men experience hair regrowth after 3–6 months^8,9^. Minoxidil must be applied for an indefinite period for continuous care of existing hair follicles and the repair of any encountered hair regrowth^6,10^.

Minoxidil, like all medications, has common side effects that are generally minor and in many cases are well tolerated. Depending on the formulation, these effects include: burning sensation, skin irritation at or near the treated area, as well as unwanted hair growth on different parts of the body have been reported^11,12^. Minoxidil sulphate is a potassium channel opener, causing hyperpolarization of cell membranes. Additionally, it can widen the blood vessels and open the potassium channels, thus allowing more oxygen, blood, and nutrients to the follicles^13^. The mechanism might promote follicles in the telogen phase to shed, which are then replenished by thicker hairs in a new anagen phase. Dose-response studies showed that minoxidil sulphate is more potent (nearly 14 folds) than minoxidil in stimulating hair follicles^6^.

Recently, there are varieties of cosmetics fibers that can conceal the thin hair areas but without any therapeutic value. In addition, most minoxidil formulations have disadvantages including instability, and messy application of the available liquid formula as well as its high coast^11,12^. Therefore, this research aims to prepare a colored nanofiber loaded with minoxidil sulphate that with constant use will help regrow hair and at the same time offers easy application of solid stable formula that will conceal the thin area during restoring hair regrowth of this area.

## Results and Discussion

### Morphology of nanofibers

**Figure 1:**
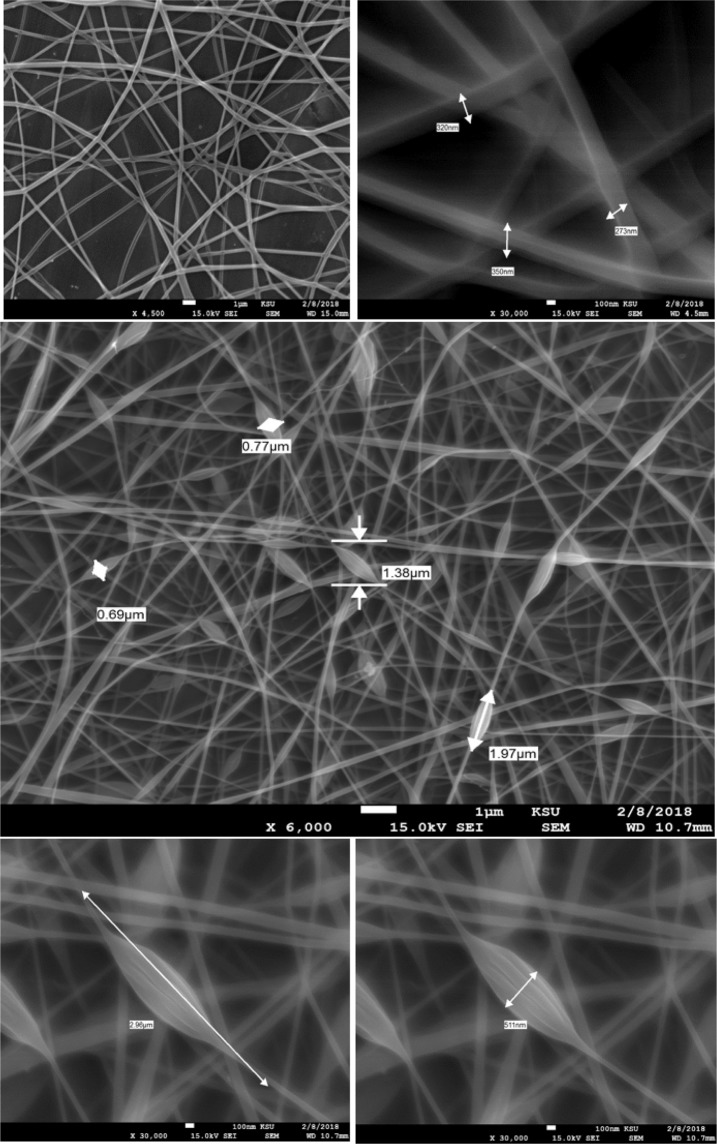
SEM images of blank fibers of 5% PVA (A and B) and minoxidil sulfate loaded nanofiber (C) showing the beads distributed along the mat and an enlarges X 30,000 bead that are filled with minoxidil sulfate (D and E) showing 511 nm width and 2.96 µm length.

The optimized fibers with desirable characteristics (average diameter of 273 nm) were produced by electrospinning of PVA solution using 5% polymer concentration under an applied electric potential of 15 kV and over a TCD of 20 cm. High density of the fiber, along with the formation of beads at regular intervals. No droplet formation observed at flow rates increased to 25 µl/min. This finding clearly indicated that with an increase in flow rate, at a high electrical force, and low PVA concentration, resulting in the stream formation of fibers and no drops observed. The brown color added to the polymeric solution and subjected to elecrtospun showed a faint color, that’s why more color powder was added after drying the fibers to achieve the required color to provide the desirable concealing to the applied area. Figure 1 showed SEM images of blank (PVA) and minoxidil sulphate-loaded nanofibers, respectively.

### Nanofibers specifications

**Figure 2:**
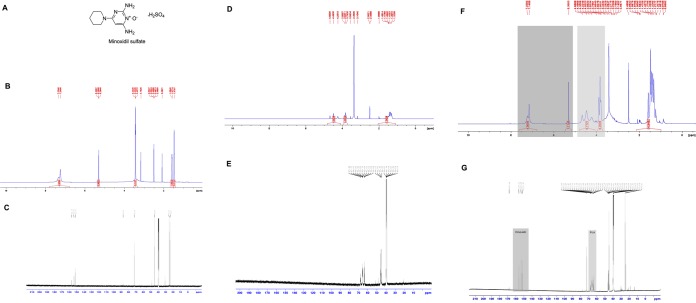
NMR spectra: ^1^H and ^13^C respective NMR spectra of pure minoxidil sulphate (**B** and **C**), and for PVA nanofiber (**D**,**E**). ^1^H NMR peaks of minoxidil sulphate were observed in the loaded PVA (**F**), ^13^C NMR peaks of minoxidil sulphate were observed in the loaded PVA (**G**). (**A**) Is the chemical structure of minoxidil sulfate.

The chemical structure of minoxidil sulfate and the NMR spectra of pure minoxidil sulphate, PVA polymer and electrospun nanofibers both blank (PVA nanofibers) and loaded with minoxidil sulphate are shown in Fig. 2(A–F, respectively). NMR spectroscopy was used to confirm the encapsulation of the drug by PVA nanofiber. ^1^H and ^13^C NMR spectra of pure minoxidil sulphate Fig. 2(B,C) and PVA nanofiber Fig. 2(D,E) were obtained and compared with the minoxidil-loaded PVA nanofiber. The following ^1^H NMR peaks of minoxidil sulphate were observed in the loaded ^1^H NMR spectrum Fig. 2(F): 1.47, 1.58, 3.43, 5.30, 7.25 and 7.33. Moreover, the ^13^C NMR peaks of the drug 24.01, 25.09, 44.88, 72.26, 155.97, 153.61 and 156.74 were detected in the loaded nanofiber spectrum Fig. 2(G). Similarly, ^1^H NMR spectrum of the pure PVA showed that the characteristic peaks of methylene (1.24–1.56), methine (3.74–3.94) and hydroxyl (4.18–4.74) protons were detected in the loaded nanofiber as well. In addition, the peaks of methylene (44.52–46.37) and methine (63.40–68.20) carbons in ^13^C NMR spectrum appeared in the loaded nanofiber spectrum. All of these findings confirmed that minoxidil sulphate was encapsulated successfully by PVA nanofiber.

DSC thermograms of PVA-minoxidil sulphate are depicted in Table 1. The loaded nanofibers prepared by electrospinning. DSC provides information about decomposition and changes in heat capacity, melting, or crystallization of the drug. Distinguished peaks were observed at 100.63 °C, as a characteristic peak of PVA, and minoxidil sulphate showed characteristic peak observed at 169.63 °C.

**Table 1:** Differential Scanning Calorimetry thermogram of PVA-Minoxidil sulphate.

Sample	DSC results
Pure minoxidil sulphate	
Blank nanofiber	
Loaded Nanofiber with Minoxidil sulphate	

The DSC of loaded nanofiber depicted the presence of minoxidil sulphate in the nanofibers; indicating stable nanofibers with no interaction between the drug and the PVA. Furthermore, the peaks have also shown not only a stable polymer, but also confirmed the stability of the drug in the nanofibers. On the other hand, the minor decrease in the melting point of the drug in the electrospun nanofibers may be ascribed to the amorphous state of the drug. The distinguished peak of the drug and polymer further indicate that there is no chemical interaction between the drug and polymer.

### HPLC analysis of minoxidil sulphate

**Figure 3:**
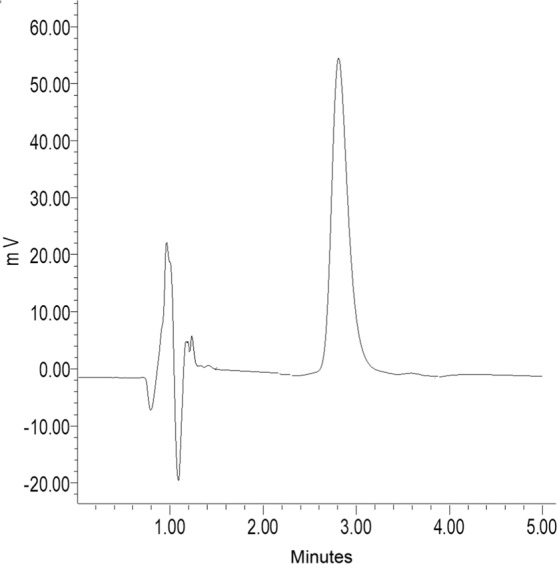
Representative chromatogram of Minoxidil sulphate (0.5 µg/ml) at 250 nm UV detection.

Selectivity is the ability of the method to distinguish and quantify the analyte in the presence of endogenous interferences. A representative chromatogram of spiked minoxidil sulphate 0.5 µg/ml is depicted in Fig. 3. The peak of minoxidil sulphate was well resolved, with retention times of approximately 1.62 min for minoxidil sulphate. The total chromatographic run time was 5.0 min.

The calibration curves were all linear with a coefficient of determination (r^2^) > 0.99 over the concentration range of 0.25–0.75 µg/ml on all tested concentrations. The calibration curve was Y = 6864.9 × −753.84 with correlation coefficient r = 0.9996, where X represents the concentration of minoxidil sulphate and Y is the area under the peak area ratio of the drug. The results implied that the method developed was linear over the specified range.

**Figure 4:**
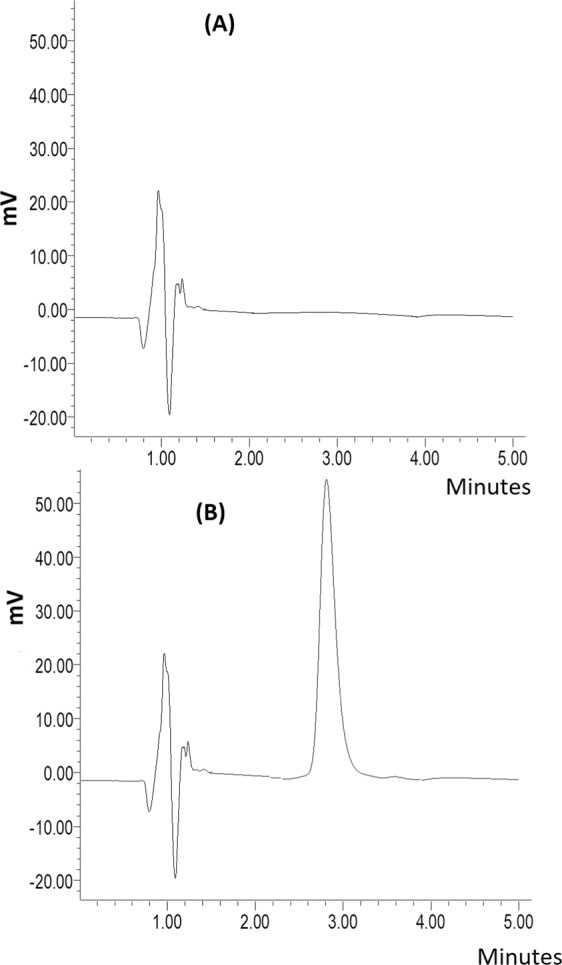
Chromatogram of minoxidil sulphate (**A**) blank sample (PVA), and (**B**) loaded minoxidil sulphate formulation at 250 nm UV detection, respectively.

Figure 4 is showing the representative chromatograms of pure sample (minoxidil sulphate (A)), sample spiked with 0.5 µg/ml minoxidil sulphate (B), while the chromatogram for formulated minoxidil sulphate nanofiber is shown in Fig. 4(C). The peak of minoxidil sulphate was well resolved, with retention times of nearly 3.28 ± 0.14 min for the drug. The total chromatographic run time was 5 min. Compared to spiked released samples, Fig. 4(A,B) showed no significant peaks, at the retention times of the drug; which proves the assay specificity. It is well observed also that the representative chromatogram of real formulation sample showed similar chromatographic behavior to quality control samples.

### Drug entrapment efficiency

The %EE of the minoxidil sulphate in the nanofiber showed minimum EE for loaded minoxidil sulphate of 47.4% which is considered suitable for delivering a therapeutically active dose. However, the yield of minoxidil sulphate-nanofiber was 73%.

### *In vitro* release study

**Figure 5:**
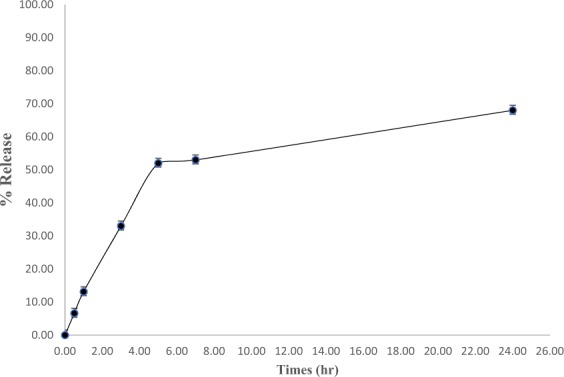
Dissolution profile of formulation at pH 6.8 (n = 3; error bars are standard deviation of n = 9).

The *in vitro* release study of minoxidil sulphate was conducted in phosphate buffer^14^ due to its ability to maintain sink conditions. The drug release pattern for 24 h is shown in Fig. 5. The profile indicated biphasic release of minoxidil sulphate from the nanofiber. The release pattern indicated that in the initial phase (5 h, r > 0.9), there was a rapid release of about 48% according to the formulation followed a slow phase from 7 to 24 h where about 20% was released. From the results obtained, it was found that there no lag time which indicates a burst in drug release during the first 15 min (about 4–10%) of the adsorbed drug on the nanofiber.

### Stability study of the prepared formulations

**Figure 6:**
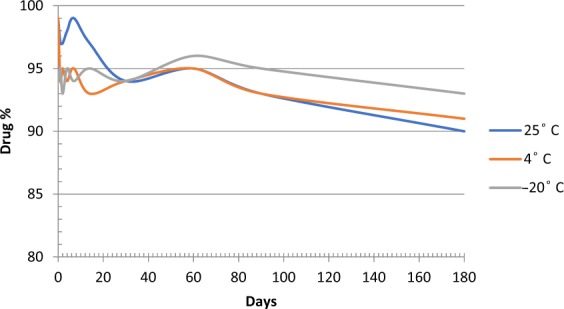
Stability of minoxidil in autosampler and after freezing– thaw cycles for 6 months (n = 9).

Minoxidil sulphate was stable in the processed samples held in the autosampler at 25 °C for 24 h with mean calculated values within 3.2% of the nominal concentration (Fig. 6). However, the samples lost only 9.6% (RSD of 4.2%) of its nominal concentration within 6 months if protected from light in the autosampler. Therefore, it is stable drug formulation and can be kept at room temperature.

The freeze–thaw temperature cycles did not significantly (p > 0.05) affect the stability of Minoxidil sulphate in all three cycles, even after 72 h, with the mean calculated values within 2.3% of the nominal concentration. Expectedly, exposing minoxidil sulphate to drastic conditions revealed that Minoxidil sulphate unstable in 2 M HCl solution and in alkaline solution, losing only 54.2% (RSD of 7.3%) of its nominal value.

## Conclusion

Based on several previous studies, the applicability and efficacy of minoxidil sulphate in restoring hair growth in both male and female who suffer from alopecia is well established and proved. However, the available formulation in-use are all in liquid unstable formula, and vastly expensive. Knowing the advantage of nanofibers prepared by electrospinning and raw materials polyvinylpyrrolidone (PVP) and minoxidil are inexpensive, and solid formula generally exhibit high stability. It is believed that this work will be valuable to produce solid nanoformula of easy use and inexpensive. The resultant minoxidil sulphate nanofibers were successfully prepared with water soluble and a biocompatible polymer, polyvinyl alcohol with desirable characteristics. The fabricated nanofibers revealed an acceptable average diameter for both unloaded and loaded fibers. Additionally, the physicochemical compatibility studies of nanofibers specified compatibility between minoxidil and used polymer and the prepared nanofibers were stable at room temperature. Therefore, the designed PVA-minoxidil sulphate loaded nanofiber serves as a potential stable solid nanoformula to be used for hair restoring as previously prov and it’s shading color as beauty coverage upon application.

## Material and Methods

### Materials

Polyvinyl alcohol PVA (1700–1800) was acquired from Loba Chemie Pvt. Ltd. (Mumbai, Indi), and minoxidil sulphate was obtained from Xi’an Fengzu Biological Technology, Co., Ltd., (Xi’an, China). Powdered Food Color of brown color PF04 was ordered from AmeriColor (Placentia, CA, USA).

### Preparation of polymer solutions

The polymer solutions were prepared using a modified version of a previously reported research work in 2016 by Sharma *et al*.^15^. In brief, PVA solutions (aqueous) of 5%, was made by dissolving of PVA in 100 ml of deionized water, with stirring for 3 h using magnetic stirrer (PTFE, SterliTech Co., North Chesterfield, VA, USA). Once PVA clear solutions achieved the result mixture of polymer was named blank nanofiber. The solution was then subjected to electrospinning (NaBond Technologies Co., Ltd., ShenZhen, Guangdong, China)^2,3^. Minoxidil sulphate (5%) was prepared by dissolving the calculated amount in ethanol that was then added dropwise and mixed by stirring with the above polymeric solution and the result formula was named loaded nanofiber.

### Preparation of nanofiber

Two different subsets of solutions were prepared and subjected to electrospinning, (a) 5% PVA, and (b) 5% PVA to be loaded with 5% minoxidil sulphate as reported^15^. The solutions were put into a 5-ml syringe fitted to a needle with a tip diameter of 22 gauges, and the syringe was then placed in the electrospinning apparatus. The polymeric solution at flow rate of 25 µl/min (voltage of 15 kV) was delivered using a syringe pump. The collector was covered with an aluminum foil of (23 cm $\ast$ 24 cm) on which the nanofibers were collected. After collection, the yielded nanofibers were dried overnight at room temperature.

### Coloring the nanofiber

The food color used does not contain any nuts products and was choose as it is safe and approved for human use. The color agent was mixed with polymeric solution and also added to the final solid fibers.

### Electrospun nanofibers characterization

#### Morphological evaluation

The size and shape of the electrospun fibers were determined by scanning electron microscopy (SEM) JSM-5510 (Jeol Ltd., Tokyo, Japan) equipped with a digital camera, at 15 kV accelerating voltage. Fibers were examined in different positions to estimate the electrospun fibers diameters^15^. A segment of the fibers was positioned on the sample holder, then sputter-coated with platinum palladium (Au/Pd) using a vacuum evaporator (Edwards)^15,16^.

#### Physicochemical compatibility studies of nanofibers

^1^H and ^13^C and NMR spectra were recorded in deuterated dimethyl sulfoxide (DMSO-d6) which was obtained from Cambridge Isotope Laboratories on a Bruker 700 MHz NMR spectrometer (Cambridge Isotope Laboratories, Inc., Tewksbury, MA, USA) using tetramethylsilane as an internal standard. Bruker Topspin software (Bruker BioSpin Corporation, Billerica, MA, USA) was used to analyze the NMR spectra generated^17^.

#### Differential scanning calorimetry of minoxidil-loaded nanofibers

Minoxidil-loaded polymeric nanofibers were thermally analyzed using differential scanning calorimetry (DSC). The DSC was performed using a Perkin Elmer Diamond hyper differential scanning calorimetry (Perkin Elmer, USA). The samples were loaded in the DSC pan and sealed, then heated from 25 to 350 °C at a rate of 10 °C/min under the flow of nitrogen gas. Melting temperature was determined from the heating curve^15,18^.

### High performance liquid chromatography (HPLC) assay for minoxidil sulphate

A modified HPLC method for minoxidil published by Rudrapal *et al*.^19^ was utilized in this study. Minoxidil sulphate concentration was quantified using a Waters HPLC system. The system consists of a Waters 2707 autosampler delivery system (Waters Inc., Bedford, MA, USA), and a symmetry C_18_ column (4.6 ×1.0 cm) packed with 5-µm spherical particles (Waters Inc., Bedford, MA, USA). The mobile phase contains of methanol:phosphate buffer pH 3.0 (60:40, v/v) at a flow rate of 1.0 ml/min. The mobile phase was prepared daily during the study, filtered through a 0.22-µm Millipore filter and degassed under vacuum. The volume injection was 10 µl, and the detection was performed at 250 nm at a run time of 4 min, and the HPLC system was operated at 40 °C. Data were analysed using an Empower Pro chromatography manager data collection system (Waters Corporation, Milford, MA, USA).

#### Standard and sample solutions

Stock solutions containing 250 mg of drug in mobile phase were stored in 4.0 ml glass vials at −20 °C. Standard calibration curve (n = 3) ranging from 0.25, 0.357, 0.5, 0.635, and 0.75 µg/ml was prepared on a daily basis to estimate the unknown minoxidil sulphate concentration for the determination of drug entrapment efficiency and drug release. Standards were transferred to glass autosampler vials with pre-slit septum, where 10 µl was injected into the HPLC system for analysis.

#### Drug entrapment efficiency and polymeric nanofiber yield determination

Minoxidil sulphate drug content in the nanofibers were precisely determined. Weighed nanofiber (10 mg) dissolved in mixture of methanol:water (1:20 v/v). Then, the solution was stirred for 60 min to assure complete dissolution of the samples. An aliquot (1.25 ml) of the stock solution was transferred into a glass autosampler vial with pre-slit septum and injected (30 µl) into the HPLC instruments using a developed validated HPLC method^20^. The minoxidil sulphate concentration was calculated using the following equations:

$$DEE( \% w/w)=\frac{{\rm{Mass}}\,{\rm{of}}\,{\rm{recovered}}\,{\rm{minoxidil}}\,{\rm{sulphate}}}{{\rm{Intial}}\,{\rm{mass}}\,{\rm{of}}\,{\rm{drug}}\,{\rm{in}}\,{\rm{formulation}}}\times 100$$

$$Yield( \% w/w)=\frac{{\rm{Mass}}\,{\rm{of}}\,{\rm{minoxidil}}\,{\rm{sulphate}}}{{\rm{Total}}\,{\rm{mass}}\,{\rm{of}}\,{\rm{polymer}},\,{\rm{excipient}}\,{\rm{and}}\,{\rm{drug}}\,{\rm{added}}}\times 100$$

### *In vitro* drug release study

The *in vitro* release test was performed using US Pharmacopeia XXXII dissolution apparatus 1 (basket). The dissolution was performed in 900 ml of phosphate buffer pH 7.2 ± 0.1 at 75 rpm and the temperature was maintained at 37 ± 0.5 °C. A sample of Minoxidil beads equivalent to 10 mg of Minoxidil was used. At appropriate time intervals (0.5, 1, 3, 5, 7 and 24 h), 2.0 ml samples were withdrawn from each vessel, mixed with 5.0 ml of methanol: water (75:25)^14,20–22^. The solution was filtered through a 0.22 µm Millipore membrane filter and analyzed using HPLC assay^20^. The volume was replaced each time with 2 ml of fresh medium and was kept at 37 ± 0.5 °C to maintain a sink condition^21^.

### Stability study of released minoxidil sulphate from the formulation

Stability studies (freeze-thaw) of the quality control samples were assessed by exposing samples to different cycles, namely: three freeze (−20 °C) and thaw (room temperature) after 0, 72 h, 1 week, 2 weeks, 4 weeks, 3 months as well as after 6 months of preparation (nanofibers), which were stored at −20 °C. The processed samples were kept in vials sealed with parafilm at 25 °C. Similarly, minoxidil sulphate was exposed to a drastic condition, in an amber volumetric glass, by diluting it in water, 1 M solution of NaOH and 2 M HCl solution (n = 6). The area under the peak (AUP) of minoxidil sulphate was measured as the zero AUP. Each solution was carefully heated to boiling and left to cool down and minoxidil sulphate AUP after boiling was measured^15,19^.
